# Peroxisome Proliferator-Activated Receptor *γ* Induces the Expression of Tissue Factor Pathway Inhibitor-1 (TFPI-1) in Human Macrophages

**DOI:** 10.1155/2016/2756781

**Published:** 2016-12-27

**Authors:** G. Chinetti-Gbaguidi, C. Copin, B. Derudas, N. Marx, J. Eechkoute, B. Staels

**Affiliations:** ^1^Inserm, CHU Lille, Institut Pasteur de Lille, U1011, EGID, Université de Lille, 59000 Lille, France; ^2^CHU, CNRS, Inserm, IRCAN, Université Côte d'Azur, Nice, France; ^3^Department of Cardiology, RWTH Aachen University, Aachen, Germany

## Abstract

Tissue factor (TF) is the initiator of the blood coagulation cascade after interaction with the activated factor VII (FVIIa). Moreover, the TF/FVIIa complex also activates intracellular signalling pathways leading to the production of inflammatory cytokines. The TF/FVIIa complex is inhibited by the tissue factor pathway inhibitor-1 (TFPI-1). Peroxisome proliferator-activated receptor gamma (PPAR*γ*) is a transcription factor that, together with PPAR*α* and PPAR*β*/*δ*, controls macrophage functions. However, whether PPAR*γ* activation modulates the expression of TFP1-1 in human macrophages is not known. Here we report that PPAR*γ* activation increases the expression of TFPI-1 in human macrophages in vitro as well as in vivo in circulating peripheral blood mononuclear cells. The induction of TFPI-1 expression by PPAR*γ* ligands, an effect shared by the activation of PPAR*α* and PPAR*β*/*δ*, occurs also in proinflammatory M1 and in anti-inflammatory M2 polarized macrophages. As a functional consequence, treatment with PPAR*γ* ligands significantly reduces the inflammatory response induced by FVIIa, as measured by variations in the IL-8, MMP-2, and MCP-1 expression. These data identify a novel role for PPAR*γ* in the control of TF the pathway.

## 1. Introduction

Macrophages are heterogeneous cells displaying a spectrum of functional phenotypes ranging from M1 proinflammatory to M2 anti-inflammatory, depending on their microenvironment [1]. Macrophages play crucial roles in the pathogenesis of atherosclerosis. Indeed, within the atherosclerotic plaque, macrophages control the inflammatory response, lipid handling (cholesterol accumulation, trafficking, and efflux) and efferocytosis [2–4]. Moreover, macrophages are also involved in atherosclerotic plaque thrombogenicity by their ability to produce both tissue factor (TF) and its natural inhibitor TFPI-1 [5, 6].

TF is a transmembrane glycoprotein member of the cytokine receptor superfamily acting as the key factor in the initiation of the blood coagulation cascade [7]. TF is expressed by endothelial cells and monocytes/macrophages after stimulation with oxidized low-density lipoproteins, lipopolysaccharide (LPS), or tumor necrosis factor (TNF)*α* [8]. Inappropriate expression of TF within the vasculature upon atherosclerotic plaque rupture leads to interaction with circulating FVIIa resulting in the formation of the TF/FVIIa complex that initiates the extrinsic coagulation pathway through a cascade of enzymatic reactions driving the conversion of FX to FXa and the production of thrombin, ultimately leading to thrombosis [9].

Beside its functions in haemostasis, the TF/FVIIa complex also plays a major role in cell migration, metastasis, and angiogenesis, probably through intracellular signalling events [10, 11]. Indeed, the TF/FVIIa complex leads to the generation of proinflammatory cytokines, such as IL-6 and IL-8 [12, 13]. The TF/FVIIa-mediated extrinsic coagulation pathway is inhibited by the tissue factor pathway inhibitor-1 (TFPI-1), a Kunitz-type inhibitor which prevents generation of FXa [8]. TFPI-1 is mainly synthesized by vascular endothelium and macrophages and is also present in plasma as free form or associated with lipoproteins or platelets [8]. The imbalance between TF and TFPI-1 ratio will thus impact both the TF/FVIIa-mediated coagulation and inflammation.

The peroxisome proliferator-activated receptor gamma (PPAR*γ*), together with PPAR*α* and PPAR*β*/*δ*, belongs to a family of transcription factors expressed in macrophages where they control the inflammatory response, cholesterol metabolism, and phagocytosis [14, 15]. PPARs also regulate macrophage thrombogenicity; indeed, PPAR*α* ligands reduce LPS-induced expression of TF [16, 17] whereas the role of PPAR*γ* in the control of TF expression is less clear; in some reports PPAR*γ* is described as having no effect [17] while others showed PPAR*γ* to decrease TF expression [18]. However, no data are available regarding the regulation of TFPI-1 expression by PPAR*γ* in human macrophages.

## 2. Materials and Methods

### 2.1. Cell Culture

Monocytes were isolated by density gradient centrifugation from healthy volunteers and differentiated into macrophages by 7 days of culture in RPMI1640 medium (Invitrogen, France) supplemented with gentamicin (40 *μ*g/mL), L-glutamine (2 mM) (Sigma-Aldrich, France), and 10% human serum (Abcys, France) [19]. M2 macrophages were obtained by differentiating monocytes in the presence of human IL-4 (15 ng/mL, Promocell, Germany), while M1 macrophages were obtained by activating differentiated macrophages with LPS (100 ng/mL, 4 h) [20]. Where indicated, synthetic ligands for PPAR*γ* (GW1929, 600 nM or rosiglitazone, 100 nM), for PPAR*α* (GW647, 600 nM), and for PPAR*β*/*δ* (GW1516, 100 nM) were added for 24 h to differentiated macrophages. Some experiments were performed on differentiated macrophages which were activated for 24 h with GW1929 (600 nM), washed, and subsequently treated in the absence or in the presence of activated FVII (FVIIa, 10 nM, Cryoprep) for further 24 h.

### 2.2. RNA Extraction and Analysis

Total cellular RNA was extracted using Trizol (Life Technologies, France). RNA was reverse transcribed and cDNAs were quantified by Q-PCR on a MX3000 apparatus (Stratagene) using specific primers (Table 1). mRNA levels were normalized to those of cyclophilin. The relative expression of each gene was calculated by the ΔΔCt method, where ΔCt is the value obtained by subtracting the Ct (cycle threshold) value of cyclophilin from the Ct value of the target gene. The amount of target relative to the cyclophilin mRNA was expressed as 2^−(ΔΔCt)^.

### 2.3. In Vivo Study

Forty nondiabetic patients after coronary stent implantation were treated with pioglitazone (30 mg daily for 8 weeks) (Supplemental Table  1 available online at http://dx.doi.org/10.1155/2016/2756781) [21]. RNA was extracted from peripheral blood mononuclear cells (PBMC) using the Paxgene Blood RNA system at both the beginning of the study and at eight-week follow-up.

### 2.4. Protein Extraction and Western Blot Analysis

After washing in cold PBS, cells were harvested in cold lysis buffer (RIPA). Cell homogenates were collected by centrifugation and protein concentrations determined using the BCA assay (Pierce Interchim). Protein lysate (20 *μ*g) was resolved by 10% SDS-PAGE, transferred to nitrocellulose membranes (Amersham), and then revealed with rabbit monoclonal antibody against TFPI-1 (Abcam) or goat polyclonal antibody against *β*-actin (Santa Cruz Biotechnology). After incubation with a secondary peroxidase-conjugated antibody (Santa Cruz Biotechnology), immunoreactive bands were revealed by chemiluminescence ECL detection kit (Amersham) and band intensity was quantified using the Quantity One software.

### 2.5. Measurement of TFPI-1 and MCP-1 Secretion by ELISA

Amounts of TFPI-1 protein were measured in culture media of macrophages treated for 24 h with GW1929 (600 nM) in the absence or in the presence of unfractionated heparin (1 U/mL, Sanofi Aventis, added 1 h before medium collection) [22], using the human TFPI Quantikine ELISA kit (R&D systems). MCP-1 secretion was measured by ELISA (Peprotech, France) according to the manufacturer's instructions.

### 2.6. Measurement of TFPI-1 Specific Activity

TFPI-1 specific activity was measured using the Actichrome TFPI activity assay (American Diagnostica) following the manufacturer's instructions in culture medium of cells treated or not for 24 h with GW1929 (600 nM).

### 2.7. Short-Interfering (si)RNA Transfection and Adenoviral Infection

Differentiated RM macrophages were transfected with siRNA specific for human PPAR*γ* and nonsilencing control scrambled siRNA (Ambion), using the transfection reagent DharmaFECT4 (Dharmacon). After 16 h, cells were incubated with GW1929 (600 nM) or vehicle (DMSO) and harvested 24 h later. For adenoviral infection, macrophages were infected with recombinant adenovirus coding for GFP (Green Fluorescent Protein, Ad-GFP) or for PPAR*γ* (Ad-PPAR*γ*) as described [23, 24]. After 16 h of infection, cells were incubated for further 24 h in the absence or in the presence of rosiglitazone (Rosi, 100 nM).

### 2.8. ChIP-seq Data Processing and Analysis

Chromatin immunoprecipitation followed by high-throughput sequencing (ChIP-seq) was performed to monitor H3K9ac levels in M2 macrophages using an antibody against H3K9ac (Millipore (17-658)) [25]. ChIP-seq data were mapped to Hg18 and signals were normalized to the total number of tags before visualization using the Integrated Genome Browser (IGB) [26]. PPAR*γ* ChIP-seq data from human primary adipocytes were obtained from [27] and PPAR*γ* response elements (PPRE) were searched using Dragon PPAR Response Element (PPRE) Spotter v.2.0 (http://www.cbrc.kaust.edu.sa/ppre/).

### 2.9. Statistical Analysis

Statistical differences between groups were analyzed by Student's *t*-test and considered significant when *p* < 0.05.

## 3. Results

### 3.1. PPAR*γ* Activation Increases the Expression and Secretion of TFPI-1 in Primary Human Macrophages

**Figure 1:**
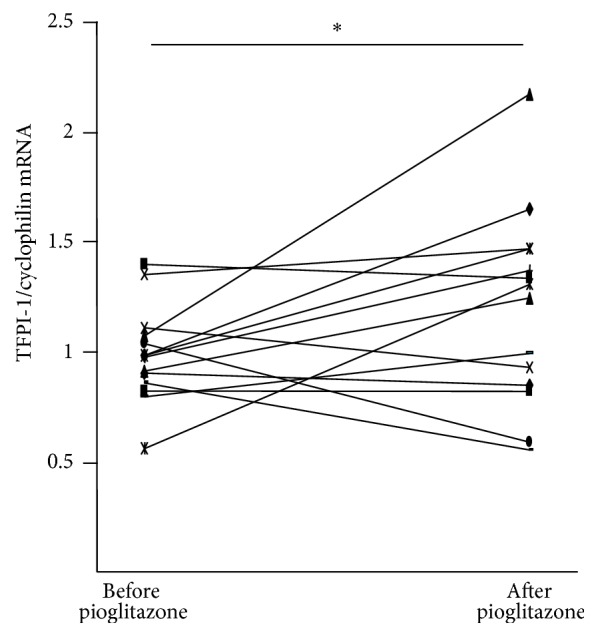
PPAR*γ* activation induces TFPI-1 expression in human blood mononuclear cells in vivo. RNA was extracted from PBMC isolated from 14 patients before and after 2 months of pioglitazone treatment (30 mg/day). TFPI-1 mRNA levels were measured by Q-PCR and normalized to cyclophilin mRNA. Statistically significant differences are indicated (*t*-test; ^*∗*^*p* < 0.05).

To investigate whether PPAR*γ* activation regulates TFPI-1 expression, peripheral blood mononuclear cells (PBMC), a cell population including circulating monocytes, were isolated from patients before and after pioglitazone administration. Interestingly, pioglitazone treatment significantly increased the expression of TFPI-1 mRNA in PBMC (Figure 1).

**Figure 2:**
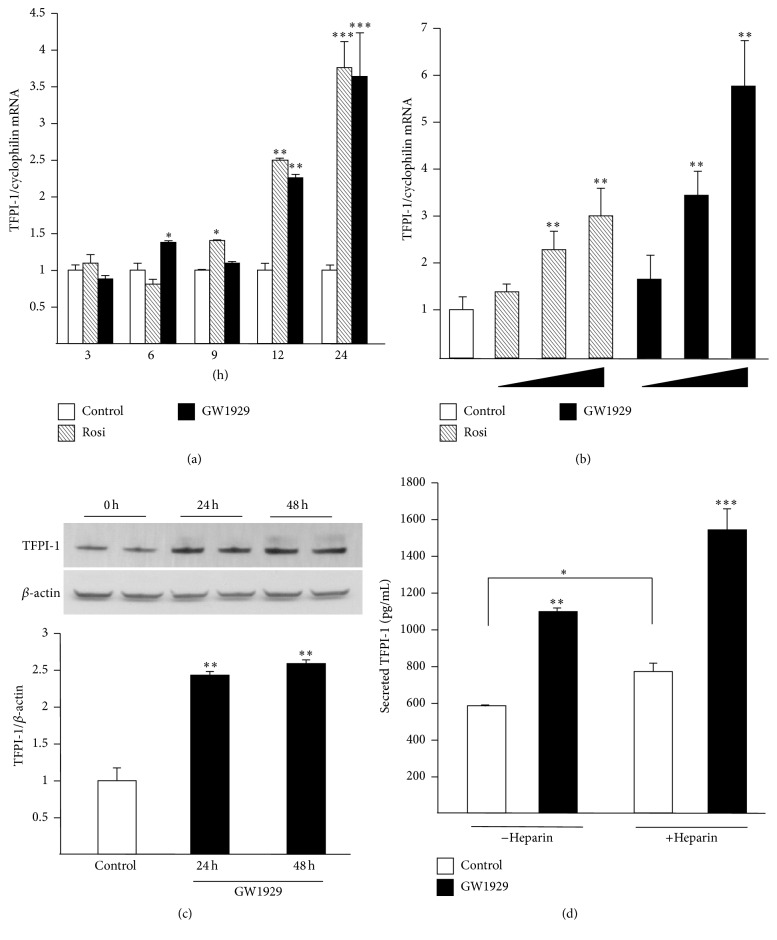
Expression of TFPI-1 is enhanced by PPAR*γ* activation in primary human macrophages. Differentiated macrophages were treated in the absence or in the presence of GW1929 (600 nM) and rosiglitazone (Rosi, 100 nM) for 3 h, 6 h, 9 h, 12 h, or 24 h (a) or with increasing concentrations of Rosi (50 nM, 100 nM, and 1 *μ*M) or GW1929 (300 nM, 600 nM, and 3 *μ*M) for 24 h (b). Total RNA was extracted and TFPI-1 mRNA levels were measured by Q-PCR and normalized to those of cyclophilin. (c) Differentiated macrophages were treated with GW1929 (600 nM) for 24 h and 48 h and TFPI-1 protein expression analyzed by western blot. TFPI-1 bands intensity was measured and normalized to those of *β*-actin. (d) Differentiated macrophages were treated with GW1929 (600 nM) in the absence or in the presence of heparin (1 U/mL), as described above. Culture media were collected and TFPI-1 protein release measured by ELISA. Results are expressed as the mean value ± SD of triplicate determinations, representative of three independent experiments. Statistically significant differences are indicated (^*∗*^*p* < 0.05, ^*∗∗*^*p* < 0.01, and ^*∗∗∗*^*p* < 0.001).

Moreover, activation of human primary differentiated macrophages with the synthetic PPAR*γ* ligands GW1929 and rosiglitazone (Rosi) resulted in the induction of TFPI-1 gene expression in a time and dose-dependent manner (Figures 2(a) and 2(b)). This regulation also occurred at the protein level in macrophages treated for 24 h or 48 h with GW1929 (600 nM) (Figure 2(c)). Induction of TFPI-1 gene expression was also observed upon PPAR*β*/*δ* and PPAR*α* activation by GW1516 and GW647 ligands, respectively (Supplemental Figure 1). Moreover, culture media TFPI-1 concentration was increased by PPAR*γ* activation with GW1929 both in the absence as well as in the presence of heparin, a factor known to enhance TFPI-1 release [22] (Figure 2(d)). However, TFPI-1 specific activity was not modified by PPAR*γ* activation in human macrophages (Supplemental Figure 2). Taken together these data indicate that PPAR*γ* activation in human macrophages increases expression and release of TFPI-1 without modifying its activity.

### 3.2. PPAR*γ* Activation Induces TFPI-1 Gene Expression Both in M1 and M2 Human Macrophages

**Figure 3:**
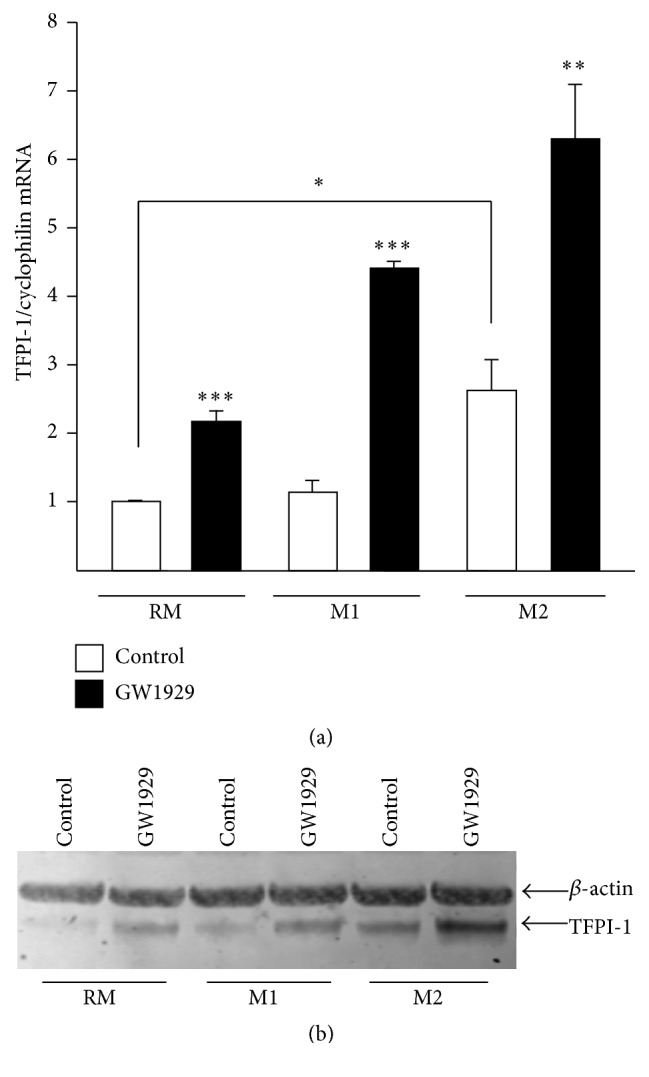
PPAR*γ* activation induces the expression of TFPI-1 in human primary macrophages irrespectively of their phenotype. Primary human monocytes were differentiated into resting unpolarized (RM) or M2 macrophages in the absence or in the presence of IL-4 (15 ng/mL) for 7 days, respectively, and then treated for 24 h with GW1929 (600 nM). M1 macrophages were obtained by activation of RM macrophages with LPS (100 ng/mL) for 4 h in the absence or in the presence of GW1929 treatment (24 h, 600 nM). (a) TFPI-1 mRNA levels were measured by Q-PCR, normalized to cyclophilin mRNA, and expressed relative to the levels in untreated cells set as 1. Results are representative of those obtained from 3 independent macrophage preparations. Each bar is the mean value ± SD of triplicate determinations. Statistically significant differences between treatment and control groups are indicated (^*∗*^*p* < 0.05; ^*∗∗*^*p* < 0.01; ^*∗∗∗*^*p* < 0.001). (b) TFPI-1 protein expression was analyzed by western blot. *β*-actin was used as loading control.

Since macrophages can present different functional phenotypes related to the microenvironment [1], the effects of PPAR*γ* activation by GW1929 were studied in nonpolarized macrophages (RM) as well as in M1 proinflammatory and in M2 anti-inflammatory macrophages. The basal expression level of TFPI-1 was significantly higher in M2 macrophages compared to both RM and M1 macrophages (Figure 3). Moreover, PPAR*γ* activation significantly induced TFPI-1 gene and protein expression in all the three different macrophage subtypes (Figure 3).

### 3.3. PPAR*γ* Ligands Regulate the TFPI-1 Expression in a PPAR*γ*-Dependent Manner

**Figure 4:**
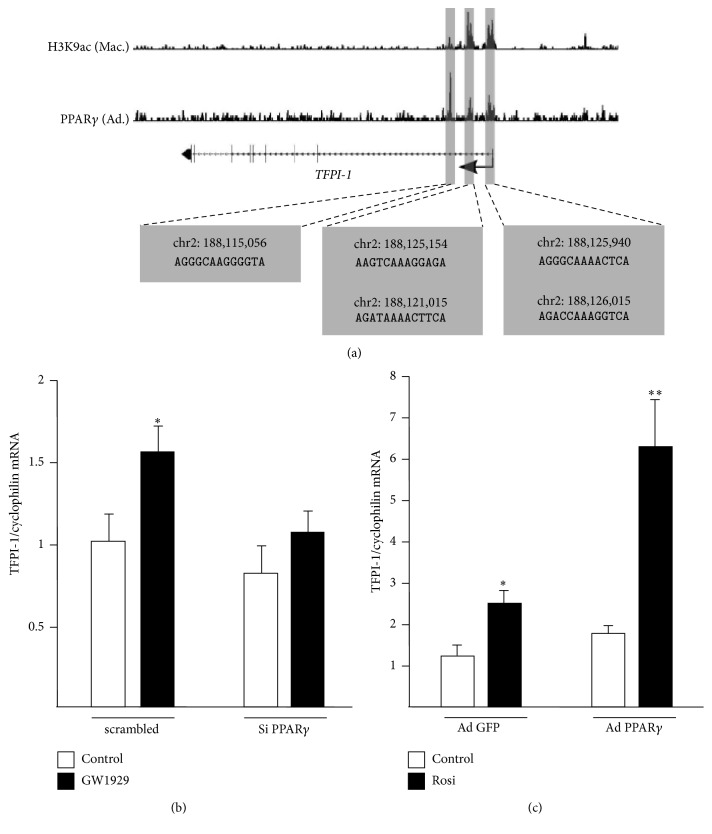
PPAR*γ* activation induces the expression of TFPI-1 in a PPAR*γ*-dependent manner. (a) H3K9ac ChIP-seq signals from M2 macrophages (Mac.) as well as PPAR*γ* ChIP-seq signal from human primary adipocytes (Ad.) are shown for the* TFPI-1* gene. Active regulatory regions are highlighted in gray and chromosomal localization (Hg18) and sequences of PPRE identified within these regions are provided at the bottom. (b) Differentiated macrophages were transfected with scrambled or human PPAR*γ* siRNA and subsequently treated with GW1929 (600 nM) or DMSO (Control) during 24 h or were infected with a GFP (Ad-GFP) or a PPAR*γ* (Ad-PPAR*γ*) adenovirus and then treated with rosiglitazone (24 h, 100 nM) (c). Statistically significant differences between treatment and control groups are indicated (^*∗*^*p* < 0.05; ^*∗∗*^*p* < 0.01).

In support of a direct regulation of* TFPI-1* gene expression by PPAR*γ*, we found that active regulatory regions encompassing or localized near the promoter of this gene, identified through enrichment for histone H3 lysine 9 acetylation (H3K9ac) in M2 macrophages, comprise putative PPAR*γ*-response elements (PPRE) and recruit PPAR*γ* in human adipocytes, a cell-type where it is highly expressed (Figure 4(a)). In order to confirm that TFPI-1 regulation induced by GW1929 treatment is due to PPAR*γ*, experiments were performed in macrophages after modulation of PPAR*γ* expression levels. The induction of TFPI-1 gene expression by GW1929 treatment was significantly reduced in the presence of the PPAR*γ* siRNA (Figure 4(b)). Complementary gain of function experiments using an adenovirus coding for PPAR*γ* (Ad-PPAR*γ*) showed that the induction of TFPI-1 gene expression by the PPAR*γ* ligand rosiglitazone was significantly enhanced in Ad-PPAR*γ*-infected macrophages, compared to Ad-GFP infected cells used as control (Figure 4(c)). These results indicate that both GW1929 and rosiglitazone activate TFPI-1 expression in a PPAR*γ*-dependent manner.

### 3.4. PPAR*γ* Activation Blocks the FVIIa-Induced Inflammatory Response in Human Macrophages

**Figure 5:**
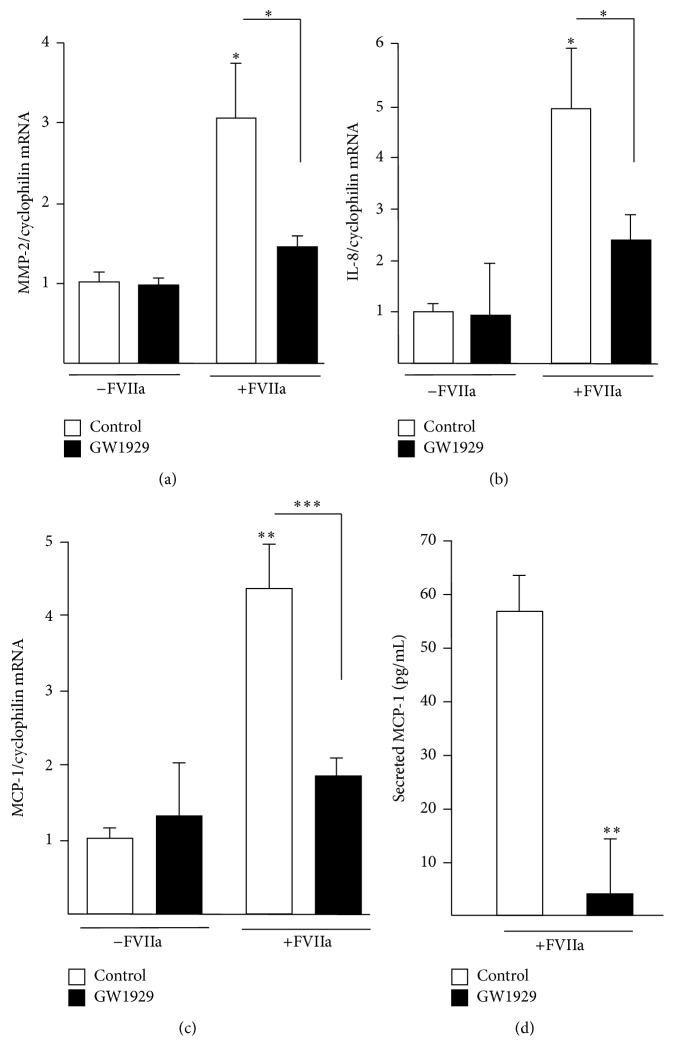
PPAR*γ* activation blocks the FVIIa-induced inflammatory response in primary human macrophages. Differentiated macrophages were treated with GW1929 (24 h, 600 nM), washed and then incubated in the absence or in the presence of FVIIa (10 nM) for further 24 h. Total RNA was extracted and MMP-2 (a), IL-8 (b), and MCP-1 (c) mRNA levels were measured by Q-PCR and normalized to those of cyclophilin. Secretion of MCP-1 was measured by ELISA in culture medium (d). Results are expressed as the mean value ± SD of triplicate determinations, representative of three independent experiments. Statistically significant differences are indicated (^*∗*^*p* < 0.05, ^*∗∗*^*p* < 0.01, and ^*∗∗∗*^*p* < 0.001).

To determine the potential biological significance of TFPI-1 induction by PPAR*γ* and given that TF/FVIIa complex can enhance an inflammatory response [12, 13], experiments were performed in macrophages treated with GW1929 (600 nM for 24 h), washed, and subsequently stimulated with FVIIa (10 nM). FVIIa induced gene expression of MMP-2, IL-8, and MCP-1, all proinflammatory molecules (Figures 5(a)–5(c)). Interestingly, treatment of macrophages with GW1929 (600 nM) significantly blocked the proinflammatory response mediated by FVIIa (Figures 5(a)–5(c)). Incubation with GW1929 also decreased FVIIa-induced secretion of MCP-1 (Figure 5(d)). These data suggest that PPAR*γ* activation can counteract the proinflammatory effects mediated by TF/FVIIa complex, the TF being expressed by macrophages [5], likely through the increase of TFPI-1 expression. Indeed, the TF/TFPI-1 ratio was significantly reduced in the presence of the PPAR*γ* agonist (Supplemental Figure 3), thus corroborating that PPAR*γ* activation blocks the FVIIa-induced inflammatory response.

## 4. Discussion

TF and FVIIa are key components of the coagulation cascade that lead to the formation of a fibrin clot. Within atherosclerotic plaque rupture this provokes thrombus generation, one of the major causes of acute ischemic syndromes such as myocardial infarction [28]. The TF/FVIIa complex has however other potential roles, since it is involved in mediating cell migration and metastasis as well as angiogenesis [29]. Indeed, TF/FVIIa can induce the production of proinflammatory cytokines and factors in keratinocytes and cancer cells [12, 13, 30].

The TF/FVIIa actions are blocked by the natural inhibitor TFPI-1. The presence of TFPI-1 has been reported in human atherosclerotic lesions where it is expressed by macrophages in areas physically close to those expressing TF and FVIIa [6]. This suggests that also in vivo, in human atherosclerotic plaques, TFPI-1 controls the TF-driven coagulation pathways as well as the thrombogenicity and can prevent complications associated with plaque rupture. However, an imbalanced expression of TF and TFPI-1 in atherosclerotic plaques can have consequences in thrombus formation as well as in inflammation.

Whether the transcription factor PPAR*γ* controls the TF-activated pathway as well as the expression of its inhibitor TFPI-1 has been matter of different studies leading to contradictory results. While it has been first reported that PPAR*γ* activation has no effect on LPS-induced TF expression in macrophages [17], other studies have shown an inhibitory effect by a mechanism involving the interference with the AP1 signalling pathway [18]. Moreover, expression of TFPI-1 has been shown to be induced by rosiglitazone in smooth muscle cells but not in THP1 macrophage cell line [18]. Here, we provide evidence that PPAR*γ* activation enhances gene, protein expression and release of TFPI-1 in human primary differentiated macrophages without affecting its specific activity. Interestingly, PPAR*γ* activation by pioglitazone treatment significantly increased the expression of TFPI-1 in PBMC, a heterogeneous cell population including circulating monocytes, thus suggesting that PPAR*γ* activation regulates TFPI-1 expression also in vivo. We have also demonstrated that the induction of TFPI-1 expression upon PPAR*γ* activation occurs in M1 proinflammatory as well as in M2 anti-inflammatory polarized macrophages. Moreover, we found that the basal expression level of TFPI-1 is higher in M2 macrophages compared to both unpolarized and M1 macrophages, suggesting that these M2 macrophages can play a major role in the control of plaque thrombosis and fibrin deposition. These data, generated in monocyte-derived macrophages isolated from healthy volunteers, are in agreement with those obtained in M2 macrophages isolated from atherosclerotic patients, in which the gene expression level of TFPI-1 is also higher in M2 compared to M1 macrophages [31]. The higher expression of TFPI-1 in M2 macrophages could thus contribute to their suggested beneficial role in plaque stabilization [32, 33].

Interestingly, in a rat carotid balloon injury model in vivo, characterized by increased neointima formation and TF overexpression, rosiglitazone injection enhances the expression of TFPI-1 protein in the injured arteries [18]. However, in vitro treatment of human atheroma specimens with rosiglitazone results in a reduced expression of TFPI-1 protein while treatment with pioglitazone led to an increased TFPI-1 expression [34]. These discrepant effects can be explained by the action of PPAR*γ* on other cellular components of the atherosclerotic plaques. Moreover, they have been obtained using high concentrations of the ligands (10 *μ*M for rosiglitazone and 5 *μ*M for pioglitazone, resp.) [34] that cannot guarantee a specificity of action over PPAR*γ* activation [35]. The induction of TFPI-1 expression upon stimulation by rosiglitazone and the GW1929 compounds in human macrophages are dependent on PPAR*γ* as demonstrated here in PPAR*γ* silencing or overexpression experiments.

Finally, we report that PPAR*γ* preactivation of macrophages significantly reduced the FVIIa-driven inflammatory response, an effect that can be mediated at least partially by the induced TFPI-1 production by PPAR*γ*.

## 5. Conclusions

In conclusion, we describe a novel function for PPAR*γ* in human macrophages in the control of the TF pathway via the induction of TFPI-1 expression, a regulation that can impact both the thrombogenicity of the atherosclerotic plaques as well as the inflammatory status induced by the TF/FVIIa complex.

## Supplementary Material

Supplemental Table 1. Baseline parameters of patients. Data are mean ± SD, *n* or median (interquartile range).
Supplemental Figure 1. PPAR*α* and PPR*β/δ* activation induces the expression of TFPI-1 in human primary macrophages. Expression of TFPI-1 was measured by Q-PCR in differentiated macrophages treated in the absence or in the presence of GW1516 (100 nM), GW647 (600 nM) or GW1929 (600 nM), for 24 h. Results are representative of those obtained from 3 independent macrophage preparations and are expressed relative to the levels in untreated cells set as 1. Each bar is the mean value ± SD of triplicate determinations. Statistically significant differences between treatments and controls are indicated (^*^p < 0.05; ^**^p < 0.01).
Supplemental Figure 2. PPAR*γ* activation does not modify TFPI-1 activity in human primary macrophages. TFPI-1 specific activity was measured in differentiated macrophages treated or not with GW1929 (600 nM) for 24 h.
Supplemental Figure 3. PPAR*γ* activation reduces the TF/TFPI-1 ratio. Differentiated macrophages were treated with GW1929 (24 h, 600 nM), washed and then incubated in the presence of FVIIa (10 nM) for a further 24 h. TF and TFPI-1 mRNA levels were measured by Q-PCR and normalized to those of cyclophilin, and their ratio calculated and expressed as the mean value ± SD of triplicate determinations. Statistically significant differences are indicated (^*^p < 0.05).

## Figures and Tables

**Table 1:** Sequences of primers used.

Gene	Forward	Reverse
TFPI-1	AGA TGG TCC GAA TGG TTT CC	ATC CTC TGT CTG CTG GAG TGA G
IL-8	CCA CCC CAA ATT TAT CAA AGA A	CAG ACA GAG CTC TCT TCC ATC A
MCP-1	TCA TAG CAG CCA CCT TCA TTC C	GGA CAC TTG CTG CTG GTG ATT C
MMP-2	TAT TTG ATG GCA TCG CTC AG	GCC TCG TAT ACC GCA TCA AT
TF	ATG TGA AGC AGA CGT ACT TGG CAC G	ATT GTT GGC TGT CCG AGG TTT GTC
Cyclophilin	GCA TAC GGG TCC TGG CAT CTT GTC C	ATG GTG ATC TTC TTG CTG GTC TTG C
