# Supplementary material for: Peroxisome Proliferator-Activated Receptor γ Induces the Expression of Tissue Factor Pathway Inhibitor-1 (TFPI-1) in Human Macrophages
Source: PPAR Res. 2016 Dec 27;2016:2756781. doi: 10.1155/2016/2756781 (PMC5223051; doi:10.1155/2016/2756781)
Supplement: Supplementary file 1 — Supplemental Table 1. Baseline parameters of patients. Data are mean ± SD, n or median (interquartile range). Supplemental Figure 1. PPARα and PPRβ/δ activation induces the expression of TFPI-1 in human primary macrophages. Expression of TFPI-1 was measured by Q-PCR in differentiated macrophages treated in the absence or in the presence of GW1516 (100 nM), GW647 (600 nM) or GW1929 (600 nM), for 24 h. Results are representative of those obtained from 3 independent macrophage preparations and are expressed relative to the levels in untreated cells set as 1. Each bar is the mean value ± SD of triplicate determinations. Statistically significant differences between treatments and controls are indicated (*p < 0.05; **p < 0.01). Supplemental Figure 2. PPARγ activation does not modify TFPI-1 activity in human primary macrophages. TFPI-1 specific activity was measured in differentiated macrophages treated or not with GW1929 (600 nM) for 24 h. Supplemental Figure 3. PPARγ activation reduces the TF/TFPI-1 ratio. Differentiated macrophages were treated with GW1929 (24 h, 600 nM), washed and then incubated in the presence of FVIIa (10 nM) for a further 24 h. TF and TFPI-1 mRNA levels were measured by Q-PCR and normalized to those of cyclophilin, and their ratio calculated and expressed as the mean value ± SD of triplicate determinations. Statistically significant differences are indicated (*p < 0.05). [file 2756781.f1.pdf]

SUPPLEMENTAL TABLE AND FIGURES

Supplemental Table 1. Baseline parameters of patients

Data are mean ± SD, n or median (interquartile range).

Supplemental figure 1. PPARα and PPRβ/δ activation induces the expression of TFPI-1 in human primary macrophages.

Expression of TFPI-1 was measured by Q-PCR in differentiated macrophages treated in the absence or in the presence of GW1516 (100 nM), GW647 (600 nM) or GW1929 (600 nM), for 24h. Results are representative of those obtained from 3 independent macrophage preparations and are expressed relative to the levels in untreated cells set as 1. Each bar is the mean value ± SD of triplicate determinations. Statistically significant differences between treatments and controls are indicated (*$p < 0.05$; **$p < 0.01$).

Supplemental figure 2. PPARγ activation does not modify TFPI-1 activity in human primary macrophages.

TFPI-1 specific activity was measured in differentiated macrophages treated or not with GW1929 (600 nM) for 24h.

Supplemental figure 3. PPARγ activation reduces the TF/TFPI-1 ratio.

Differentiated macrophages were treated with GW1929 (24h, 600 nM), washed and then incubated in the presence of FVIIa (10 nM) for a further 24h. TF and TFPI-1 mRNA levels were measured by Q-PCR and normalized to those of cyclophilin, and their ratio calculated and expressed as the mean value ± SD of triplicate determinations. Statistically significant differences are indicated (*$p<0.05$).

Supplemental Table 1.

Age	63.1+2.3
Smokers	8
Sexe (male/female)	11/3
BMI	25.9 (23.7;29.4)
Fasting glucose (mmol/L)	5.3 (4.8;5.5)
Fasting insulin (µU/L)	5.6 (4.2;8.4)
Total cholesterol (mmol/L)	4.3 (4.0;4.9)
HDL cholesterol (mmol/L)	1.2 (1;1.4)
Triglycerides (mmol/L)	1.2 (1;2.6)

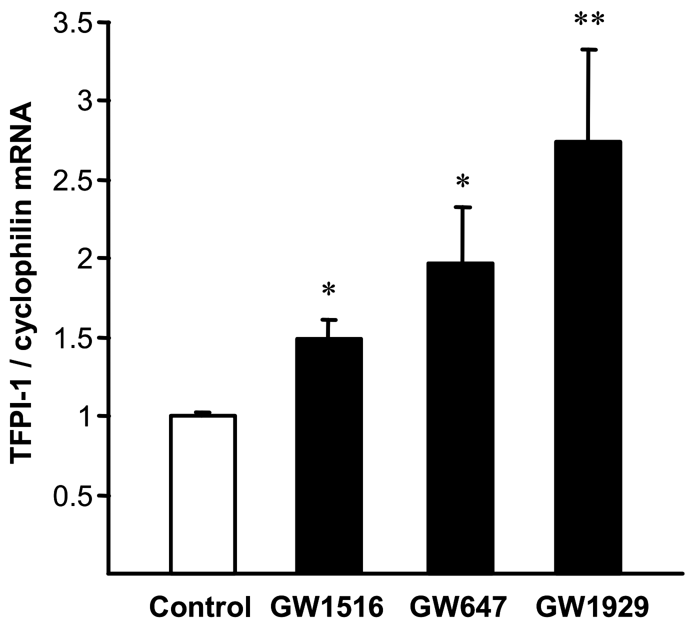

Supplemental figure 1

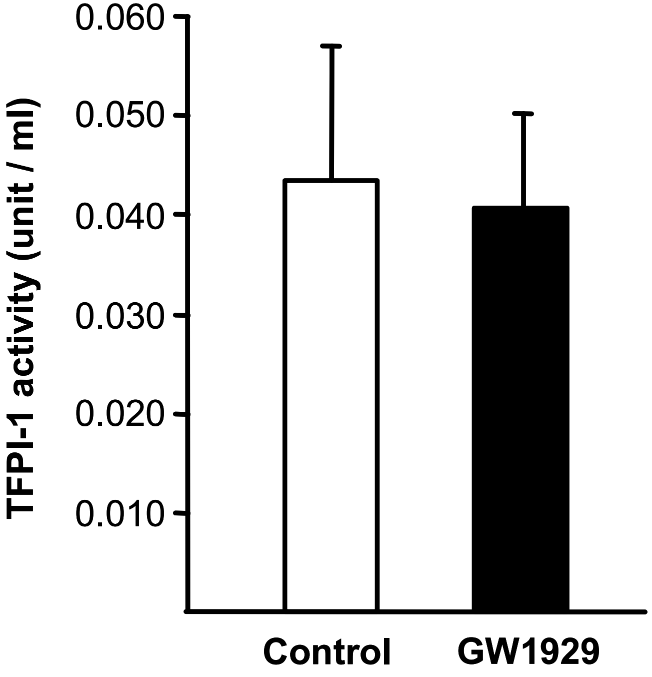

Supplemental figure 2

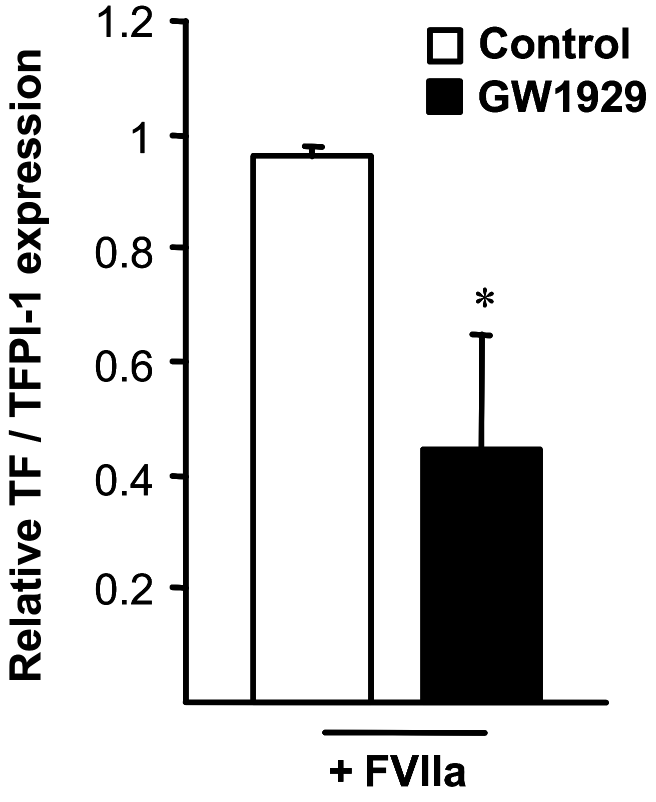

Supplemental figure 3
